# Association of Micronutrients and Handgrip Strength in Korean Older Population: A Cross-Sectional Study

**DOI:** 10.3390/healthcare10101980

**Published:** 2022-10-09

**Authors:** Na-Hyung Kim, Choon Young Kim

**Affiliations:** 1 Department of Food and Nutrition, Yeungnam University, Gyeongsan 38541, Korea; 2 Institute of Human Ecology, Yeungnam University, Gyeongsan 38541, Korea

**Keywords:** micronutrients, mineral, vitamin, potassium, handgrip strength, dynapenia, sarcopenia

## Abstract

Sarcopenia is characterized by the loss of skeletal muscle mass, strength, and physical performance. Dynapenia and kratopenia are described as the loss of muscle strength and power. Nutritional intake status is one of the factors affecting the prevention of an age-related muscle decline such as sarcopenia, dynapenia, or kratopenia in older populations. This study aimed to investigate the association between the intake of micronutrients and handgrip strength in 1254 individuals (546 men and 708 women) of the Korean older population from the most recent dataset. They were analyzed and divided into two groups: a LHS group with low handgrip strength (<28 kg for men and <18 kg for women) and a normal group with normal handgrip strength. Logistic regression analysis was performed to estimate the odds ratios (ORs) and 95% confidence intervals (Cis) of the associations between micronutrient intakes and low handgrip strength in Korean older population by gender. Among micronutrients, insufficient potassium intake showed a significant association with low handgrip strength for men (OR: 3.159, 95% CI: 1.164–8.578) and women (OR: 2.793, 95% CI: 1.380–5.654) aged ≥65 years, respectively (*p* = 0.005 for men, *p* = 0.024 for women), as a result of adjusting for all confounding factors that could affect low handgrip strength. In conclusion, potassium intake among micronutrients in Korean older populations with low handgrip strength might need continuous monitoring for the intervention or prevention of dynapenia or sarcopenia.

## 1. Introduction

Handgrip strength is a representative method for measurement of muscle strength and muscle power. The measurement of handgrip strength is a common procedure to evaluate an age-related muscle decline such as dynapenia, kratopenia, and sarcopenia in clinical practice [1,2,3]. Among these geriatric muscle decline, sarcopenia has recently been characterized by progressive and degenerative loss of skeletal muscle mass, strength, and power, as well as declining physical performance, all of which encompass the symptoms or phenomenon related to aging [4,5]. Sarcopenia was established as a muscle disease, according to the International Classification of Diseases, Tenth Revision, Clinical Modification diagnosis code M62.84, on 1 October 2016 [6]. Starting from the age of 40 years, the loss of muscle mass in individuals is reported to be approximately 1–2% per year after the age of 50 years [7]. The prevalence of sarcopenia increases with age, affecting between 5 and 13% of subjects aged 60–70 years and between 11 and 50% of those aged over 80 years [8]. “Possible sarcopenia” is defined by either low muscle strength or low physical performance only and it is specifically used in primary health care or community-based health promotion to enable earlier lifestyle interventions [9]. Moreover, dynapenia and kratopenia are known as muscle decline described as the loss of muscle strength and power [10].

The loss of muscle mass and strength increases the risk of chronic diseases such as type 2 diabetes mellitus [11], obesity [12], non-alcoholic fatty liver disease [13], osteoporosis [14,15], chronic obstructive pulmonary disease [16], cancer [17], chronic heart failure [18], and chronic kidney disease, which is associated with cardiovascular disease, stroke, and renal failure [15,19]. Considering the multi-dimensionality of sarcopenia, a previous study demonstrated that the relationship between physical activity and nutritional intake, although the mechanisms have not yet been fully clarified, is partly elucidated by the atrophy of skeletal muscle type 2 fibre and a relative elevation in type 1 fibre density, according to altered protein metabolism and dysregulated autophagy inducing muscle atrophy [20]. Additionally, previous studies have reported that individual or combined interventions with physical activity, such as resistance training or nutritional supplementation, among various factors affecting the prevention of sarcopenia, had an influence on its development in older adults [21,22]. A recent systematic review and meta-analysis on the relationship between calorie and nutrient intake and sarcopenia among older study population demonstrated that a difference in intake of caloric, macronutrients such as proteins, carbohydrates, or saturated fatty acids, and micronutrients including calcium, magnesium, sodium, selenium, vitamin A, vitamin B12, vitamin C, and vitamin D exists depending on the presence or absence of sarcopenia [23].

Although numerous studies on helpful nutrients for the prevention of dynapenia or sarcopenia are continuously being conducted, the association between several minerals or vitamins and dynapenia or sarcopenia among older populations with and without dynapenia or sarcopenia remains unclear. Thus, this study was designed to investigate the association between the micronutrient intakes and handgrip strength in Korean older individuals aged 65 years and over, by using the database from the Korea National Health and Nutrition Examination Survey, a nationwide cross-sectional cohort with a nationally representative sample that was recently collected.

## 2. Materials and Methods

### 2.1. Settings and Study Population

**Figure 1:**
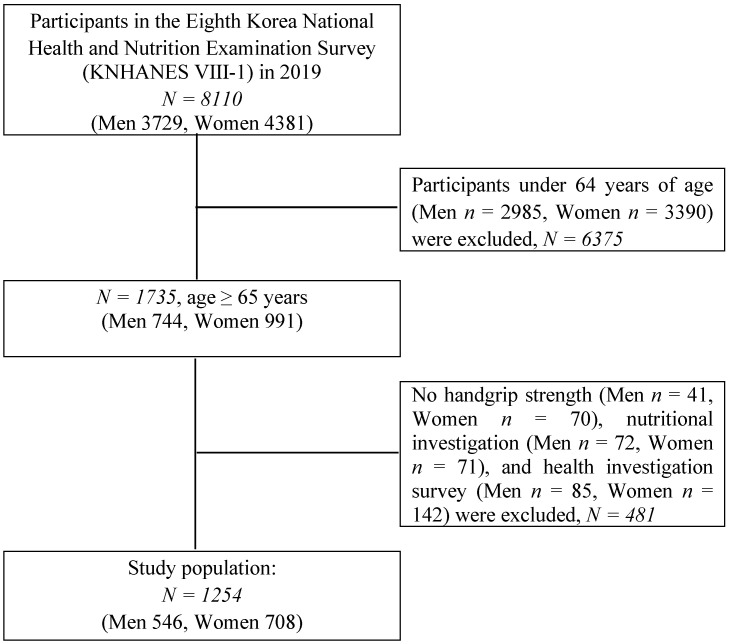
Flowchart of the study population from the Korean National Health and Nutrition Examination Survey (2019), showing the inclusion and exclusion criteria.

The Korea National Health and Nutrition Examination Survey (KNHANES), which is conducted annually by the Korea Disease Control and Prevention Agency, is a rolling sample survey and a complex multistage stratified cluster survey. It contains nationally representative and reliable statistical data, including the health level, health behaviour, food and nutritional intake status, and prevalence of chronic diseases in Koreans [24]. All survey protocols were approved by the Institutional Review Board of the Korea Disease Control and Prevention Agency (No. 2018-01-03-C-A) and were conducted in accordance with the Declaration of Helsinki. This study was categorized as an exemption by the Institutional Review Board of Yeungnam University. All participants signed an informed consent form. Of the 8110 individuals who participated in the KNHANES 2019, the following participants were excluded from the study: individuals under the age of 65 years (*n* = 6375), those with missing data on handgrip strength for the right or the left hand (*n* = 111), those with missing data on nutritional information (*n* = 143), and those without health survey data (*n* = 227). Finally, 1254 participants (546 men and 708 women) were included in this study (Figure 1).

### 2.2. Measurement of Handgrip Strength

Handgrip strength was evaluated three times per hand using a digital grip strength dynamometer T.K.K 5401 (Takei Scientific Instruments Co., Ltd., Tokyo, Japan). Prior to the examination of handgrip strength, people with arm, hand, or thumb defects, defects or fractures of other fingers except the thumb, and hand paralysis and people with casts or bandages on the hands or wrists were excluded. As described previously [25,26], trained medical technicians instructed the seated participants to hold the dynamometer with the distal interphalangeal finger joints of the hand at a 90° angle to the handle and to squeeze the handle as firmly as possible. After participants had slowly stood up, they looked straight ahead with both arms falling naturally; their elbows and wrists were not flexed, their arms were not in contact with their bodies, their feet were hip-width apart and evenly spaced, and their handgrip strength was measured for 3 s while exhaling. To reduce the effect of repetition fatigue, participants conducted the handgrip strength tests with a 60 s rest period between each attempt. Before the next attempt, the participants were checked for correct postures. The maximum of the three measured values for each respective hand, acquired from both the left and right hands or one hand, was taken as the final handgrip strength. In the present study, low handgrip strength was defined as a handgrip strength <28 kg for men and <18 kg for women according to the criteria recommended by the Asian Working Group for Sarcopenia in 2019 [9].

### 2.3. Measurement of Nutritional Intake Status

The amount of nutrient intake was analyzed from food intake survey data collected using the 24-h dietary recall method. The 24-h dietary recall was conducted by trained dietary staff, and participants were examined for all foods consumed during 24 h from when they woke up until they went to bed the previous day. All foods consumed during the 24 h from 12:00 to 12:00 the day before the survey were recorded for participants with irregular life patterns, such as night-shift workers. During the examination, the implements for food consumption including a volume calculation tool, a two-dimensional food model, bowls or dishes, and measuring cups and spoons were used to improve the accuracy of the data. Nutrient intake was calculated from the collected data using the National Standard Food Composition DB 9.0 by the Rural Development Administration National Institute of Agricultural Sciences [27]. Dietary intake was compared with the criteria of the 2020 Dietary Reference Intake for Koreans established by the Ministry of Health and Welfare and the Korean Nutrition Society [28]. We referenced the recommended nutrient intake values for calcium, phosphorus, iron, vitamin A, thiamine, riboflavin, niacin, folate, and vitamin C, and the adequate intake values were used for sodium and potassium because the recommended nutrient intake values for only sodium and potassium have not been established in the 2020 Dietary Reference Intake for Koreans [28].

### 2.4. Covariates

In the present study, we considered confounding variables such as age, household income, current smoking status, monthly alcohol consumption, aerobic exercise, muscular exercise (strengthening), comorbidities including hypertension, obesity, diabetes, and hypercholesterolemia, and total calorie, total protein, total fat, and total carboxylate intake. Household income quantiles were categorized as low, low-middle, middle-high, and high. Current smoking status was categorized as non-smokers and current smokers. Monthly alcohol consumption was categorized as participants who did not consume alcohol (‘no’) and those who had consumed at least one glass per month in the past year (‘yes’). Aerobic exercise was classified as the performance of at least 150 min of moderate-intensity physical activity per week, at least 75 min of high-intensity physical activity in a week, or a combination of moderate-intensity and high-intensity physical activity per week (‘yes’). Here, 1 min of high-intensity exercise was considered equal to 2 min of moderate intensity. Muscular exercise was classified as participation in strength training exercises such as push-ups, sit-ups, and use of dumbbells or barbells for one or more days in the last week (‘yes’). Comorbidities such as hypertension, obesity, diabetes, and hypercholesterolemia were investigated. Hypertension was classified into three levels: normal (‘no’), pre-stage hypertension, and hypertension (‘yes’). Hypertension was defined as systolic blood pressure ≥140 mmHg, diastolic blood pressure ≥90 mmHg, or use of antihypertensive medications. Pre-hypertension was defined as systolic blood pressure ≥120 mmHg and <140 mmHg or diastolic blood pressure ≥80 mmHg and <90 mmHg, and normal stage was defined as systolic blood pressure <120 mmHg or diastolic blood pressure <90 mmHg. Obesity was classified into four levels: low weight (body mass index (BMI) < 18.5), normal state (18.5 ≤ BMI < 23), pre-stage obesity (23 ≤ BMI < 25), and obesity (25 ≤ BMI) according to BMI values calculated by weight (kg) divided by height (m) squared. Diabetes was defined when participants had fasting serum sugar levels ≥126 mg/dL after 8 h of fasting, were taking anti-diabetic medications or insulin injections, had an official diagnosis, or had hemoglobin A1c ≥ 6.5%. This category was classified into three stages: normal state (fasting serum sugar < 100 mg/dL or hemoglobin A1c ≤ 5.7%, ‘no’), pre-stage diabetes (100 ≤ a fasting serum sugar ≤ 125 or 5.7 ≤ hemoglobin A1c ≤ 6.4%), and diabetes (‘yes’). According to the presence or absence of hypercholesterolemia, the subjects were divided into two groups: total cholesterol ≥240 mg/dL after 8 h of fasting or taking anti-hypercholesterolemia medications (‘yes’).

### 2.5. Statistical Analysis

Data were statistically analyzed using the IBM SPSS Statistics 25.0 analysis program (IBM Corporation, Armonk, NY, USA). Continuous and categorical variables were presented as mean ± standard error or percentage (*n* (%)), respectively. Differences in the general characteristics of the participants according to handgrip strength were compared using the *t*-test and the Rao–Scott chi-square test. Due to the significantly different handgrip strength criteria values in men and women, respectively, we divided the subjects into two groups according to sex, and the data were analyzed separately. Logistic regression analysis was performed to estimate the odds ratios (ORs) and 95% confidence intervals (CIs) of the association between micronutrient intakes and low handgrip strength. Multivariate analysis was conducted after adjusting for covariates and was initially adjusted for age and sex as model 1. Model 2 added household income, current smoking status, monthly alcohol consumption, aerobic exercise, and muscular exercise to the model 1 covariates. Model 3 included comorbidities including hypertension, obesity, diabetes, hypercholesterolemia, and macronutrient intakes such as energy intake of total calories, total protein, total fat, and total carbohydrate as model 2 covariates. A *p* value < 0.05 was considered statistically significant.

## 3. Results

### 3.1. Anthropometric, Demographic, and Clinical Data 

Of the study population (546 men, 708 women), 18.9% and 23.7% of the men and women had a low handgrip strength, respectively, per the criteria of the Asian Working Group [9]. The mean age of the men in the study population was 75.92 and 72.07 years for the LHS and normal groups, respectively. The mean age of the women in the two groups was approximately 75.27 and 71.46 years, respectively. For both men and women, the LHS group was older than the normal group (*p* < 0.001). Further characteristics of the study populations between LHS group and normal group are compared in Table 1. The men and women groups with low handgrip strength showed significant differences in household income compared to the group with normal handgrip strength (*p* < 0.001). Among men, the prevalence of aerobic exercise and muscular exercise (strengthening) in the normal group was higher than that in the low handgrip group (*p* < 0.001). The proportion of women who performed muscular exercises was higher in women of a normal group than in those with low handgrip strength (*p* = 0.034). For men, the mean maximum handgrip strengths of the LHS and normal groups were 23.69 ± 0.38 kg and 36.10 ± 0.26 kg, respectively, and these were significantly different (*p* < 0.001). Similarly, the female in the LHS group showed a lower handgrip strength value of 15.03 ± 0.20 kg in comparison to the normal group, which had a mean handgrip strength of 22.84 ± 0.18 kg (*p* < 0.001). The height, body weight, and BMI were significantly lower in the group with low handgrip strength than the normal group in both genders. However, there were no differences in comorbidities, including hypertension, diabetes, and hypercholesterolemia, among men and women when compared to the respective normal groups except obesity in women (*p* = 0.043).

### 3.2. Nutritional Risk Factors

Sufficient caloric and protein intake is known to have a positive effect on sarcopenia prevention. In the present study, the average caloric intake of the subjects with low handgrip strength was 1552.26 ± 55.78 kcal (1 kcal = 4.186 kJ), which was less than the respective estimated 2000 kcal or 1900 kcal of energy needed for men 65–74 years of age or over 75 years of age per the criteria of the 2020 Dietary Reference Intake for Koreans [28]; in addition, those with weakened muscles consumed significantly fewer calories per day than the normal group in men (*p* < 0.001). The total energy intake of women with low handgrip strength tended to be lower than that of the normal group, but the difference was not significant. For both men and women, the average protein intake was significantly lower in the LHS groups than in the normal groups (*p* = 0.001 and *p* = 0.035, respectively). In relative macronutrient intakes per day normalized by body mass (g/kg BM/day), fat intake was significantly differences between the LHS group and the normal group for men (*p* < 0.001). Regarding mineral and vitamin intake, subjects with low handgrip strength consumed significantly lower amounts of calcium, phosphorus, iron, potassium, riboflavin, niacin, folate, and vitamin C than those who did not, in both men and women. Intergroup differences in the levels of sodium, vitamin A, β-carotenoids, and thiamine intake were verified in men (*p* < 0.05), although there was no significance for women when compared with the normal group, as described in Table 2.

### 3.3. Multiple Logistic Regression for Low Handgrip Strength in Men Aged ≥ 65 Years

Table 3 outlines the results of the logistic regression analysis performed to estimate the ORs and 95% CIs of the correlations between intake of micronutrients such as minerals or vitamins and low handgrip strength in men. The insufficient intakes of calcium (OR: 2.662, 95% CI: 1.272–5.568), phosphorus (OR: 2.752, 95% CI: 1.676–4.519), and iron (OR: 2.235, 95% CI: 1.359–3.676) were associated with sarcopenia and remained significant after adjusting for covariates (model 1) (OR: 2.438, 95% CI: 1.121–5.299 for calcium, OR 2.181, 95% CI: 1.322–3.597 for phosphorus, and OR 1.723, 95% CI: 1.015–2.923 for iron). Among minerals, it only remained a significant association between insufficient potassium intake and low handgrip strength in model 3, which adjusted all covariates (OR: 3.159, 95% CI: 1.164–8.578, *p* = 0.024). The crude data, which did not adjust for any variables, of vitamin intake showed a significant association between low handgrip strength and insufficient intakes for four kinds of vitamins: 2.848 (1.029–7.882) for vitamin A, 2.882 (1.680–4.944) for thiamine, 2.555 (1.466–4.454) for riboflavin, and 2.115 (1.196–3.739) for folate. However, the association between low handgrip strength and insufficient vitamin intake in men aged ≥ 65 years after controlling for all covariates had no significant differences.

### 3.4. Multiple Logistic Regression for Low Handgrip Strength in Women Aged ≥ 65 Years

The female group with low handgrip strength was associated with insufficient intake of calcium (OR: 3.074, 95% CI: 1.271–7.437), phosphorus (OR: 1.707, 95% CI: 1.182–2.466), iron (OR: 1.686, 95% CI: 1.191–2.387), potassium (OR: 3.151, 95% CI: 1.576–6.298), and riboflavin (OR: 1.758, 95% CI: 1.120–2.760) when unadjusted. Association between the insufficient potassium intake and low handgrip strength in women remained significant after controlling for all covariates (model 3) (OR: 2.793, 95% CI: 1.380–5.654), as in the case of men (Table 4).

## 4. Discussion

In this cross-sectional study, we defined low handgrip strength according to the proposed sex-specific cut-off values of handgrip strength (<28 for men and <18 for women) established in 2019 by the Asian Working Group for Sacorpenia [9]. Through most recently dataset examined from KNHANES, although it was very limited, we used handgrip strength to evaluate skeletal muscle strength which can affect dynapenia or sarcopenia. The updated European Working Group on Sarcopenia in Older People (EWGSOP2, version 2019) defined sarcopenia as decreased appendicular skeletal muscle mass (ASM) (cut-off points: <20 kg for men, <15 kg for women), skeletal muscle mass index (SMI = ASM/height squared (kg/m^2^) (7.0 kg/m^2^ for men, <5.5 kg/m^2^ for women), and handgrip strength (<27 kg for men, <16 kg for women), respectively [29,30]. A previous study using the National Health and Nutrition Examination Surveys (NHANES) from 1999 to 2004 showed 30% of men and 8.8% of women had low muscle mass among the study participants aged 65 years and older when defined by SMI (kg/m^2^) criteria [31]. In a current cross-sectional study, it was shown that approximately 18.9% of men and 23.7% of women had low handgrip strength among the study population who were 65 years old or older according to handgrip strength (kg) with criteria of <28 kg for men and <18 kg for women although a small sample size.

Regarding the relationship between household income and sarcopenia, there have been reports stating that incomes differ significantly between the sarcopenia and non-sarcopenia groups [32]; conversely, income was not a significant factor affecting handgrip strength in another study [33]. Our results showed intergroup differences in household income for both genders. Compared with those in the normal group, the ratio of the low and middle-low class of household income was higher in the LHS group. Inconsistent with a previous study that demonstrated that male participants aged >65 years with high handgrip strength were more likely to be smokers than those with low handgrip strength [32], there was no intergroup difference for low handgrip strength or normal for both sexes in the current study. While the proportion of participants with low handgrip strength were less likely to have alcohol intake than those in the normal group in men, there was no difference between the women groups. The proportions of men in the LHS group that performed aerobic and muscular exercises and women in the LHS group that performed muscular exercises were lower than those in the normal groups. Previous studies have also published that individual or combined interventions that employ physical activities such as resistance training or encourage nutritional supplementation in older adults play a major role in prevention of sarcopenia [21,22].

Similar to the lack of association between sarcopenia and comorbidities such as hypertension, diabetes, and dyslipidemia in elderly men [32], comorbidities such as hypertension, diabetes, and hypercholesterolemia did not differ between the LHS and normal groups in this study. Among the examined comorbidities in older women, the proportion of participants with low handgrip strength in the pre-obesity and obesity groups tended to be lower than that in the normal group. A recent cohort study [34] showed that prevalence of sarcopenic obesity and sarcopenic overweight increased with age to 4.2% or 26.6% in those aged 70–79.9 years, and 12.2% or 51.1% in those aged 80–89.9 years, respectively.

With regard to the multi-dimensionality of sarcopenia, possible pathogenesis routes such as central nervous system decline or loss of gonadal steroids [5], cellular or mitochondrial dysfunction [35], cachexia [36], and malnutrition [37,38] have been reported as molecular mechanisms of sarcopenia. According to a prospective population-based cohort study in Belgium on aged 65 and over, who had sarcopenia and physical impairment due to advancing age, the prevalence of malnutrition was 23.4% according to the Global Leadership Initiative on Malnutrition criteria and 7.3% according to the European Society of Clinical Nutrition and Metabolism (ESPEN) criteria [38]. Older individuals with sarcopenia were reported to consume statistically lower amounts of calories, macronutrients such as proteins, carbohydrates, and saturated fatty acids, and micronutrients including vitamin A, vitamin B12, vitamin C, vitamin D, calcium, magnesium, sodium, and selenium, compared to those without sarcopenia [23]. As for the nutrients analyzed in our study, consistent with a previous study, the intakes of calorie, proteins, fat, carbohydrates, vitamin A, vitamin C, calcium, and sodium were lower in older men with low handgrip strength than in those of normal group. Moreover, both men and women showed a decrease in intakes of total protein in the group with low handgrip strength, compared with the normal group. The associations between total energy or total protein intake and the prevalence of sarcopenia are related to the mechanisms through atrophy of skeletal muscle type 2 fibre and a relative elevation in type 1 fibre density according to an altered protein metabolism and a dysregulated autophagy inducing muscle atrophy [5,20]. Housteon et al. reported that energy-adjusted protein intake was associated with 3-year changes in total lean mass [β (SE): 8.76 (3.00), *p* = 0.004] and non-bone appendicular lean mass [β (SE): 5.31 (1.64), *p* = 0.001] in men and women aged 70–79 years, after adjustment for potential confounders [39]. Since the initial aim of the current study was to examine the associations between micronutrients including several minerals or vitamins and low handgrip strength, we did not express the macronutrients as a percentage, using the acceptable macronutrient distribution ranges. Among micronutrients investigated in this study, the levels of calcium, phosphorus, iron, sodium, potassium, vitamin A or its derivatives, β-carotenoids, and thiamine, riboflavin, niacin, and folate, known as kinds of vitamin B.

**Table 4:** Multiple logistic regression for low handgrip strength in women aged ≥ 65 years.

Regression Models	Reference	Unadjusted			Model 1			Model 2			Model 3		
		OR	95% CI	*p* Value	OR	95% CI	*p* Value	OR	95% CI	*p* Value	OR	95% CI	*p* Value
Calcium intake (<800 mg)	≥800 mg	3.074	1.271–7.437	**0.013**	2.521	0.984–6.454	0.054	2.213	0.881–5.560	0.090	2.415	0.841–6.938	0.101
Phosphorus intake (<700 mg)	≥700 mg	1.707	1.182–2.466	**0.005**	1.482	1.020–2.156	**0.039**	1.351	0.906–2.017	0.139	1.660	0.794–3.470	0.176
Iron intake (<8 mg)	≥8 mg	1.686	1.191–2.387	**0.004**	1.345	0.929–1.948	0.116	1.237	0.830–1.842	0.294	1.241	0.644–2.390	0.517
Sodium intake (≥1300 mg)	<1300 mg	1.123	0.744–1.695	0.579	1.074	0.702–1.643	0.742	0.986	0.638–1.523	0.947	0.904	0.559–1.463	0.680
Potassium intake (<3500 mg)	≥3500 mg	3.151	1.576–6.298	**0.001**	2.554	1.291–5.051	**0.007**	2.320	1.175–4.581	**0.016**	2.793	1.380–5.654	**0.005**
Vitamin A intake (<600 µg RAE)	≥600 µg RAE	1.162	0.596–2.266	0.657	1.064	0.544–2.080	0.855	0.930	0.478–1.812	0.830	0.855	0.405–1.802	0.678
Thiamine intake (<1.0 mg)	≥1.0 mg	1.097	0.729–1.650	0.655	0.867	0.569–1.319	0.502	0.826	0.539–1.266	0.378	0.720	0.399–1.299	0.273
Riboflavin intake (< 1.1 mg)	≥1.1 mg	1.758	1.120–2.760	**0.015**	1.336	0.829–2.153	0.232	1.155	0.698–1.911	0.573	1.388	0.729–2.643	0.316
Niacin intake (<13 mg)	≥13 mg	1.394	0.792–2.455	0.248	1.053	0.561–1.978	0.872	0.943	0.490–1.815	0.860	0.716	0.306–1.676	0.439
Folate intake (<400 µg DFE)	≥400 µg DFE	1.273	0.771–2.104	0.343	1.125	0.681–1.859	0.643	1.058	0.639–1.753	0.825	0.940	0.547–1.614	0.822
Vitamin C intake (<100 mg)	≥100 mg	1.788	0.993–3.218	0.053	1.485	0.817–2.700	0.193	1.353	0.729–2.511	0.336	1.257	0.623–2.535	0.520

Dietary intake was compared with the Korea Dietary Recommendation Intake criteria. Recommended intake was used for calcium, phosphorus, iron, potassium, vitamin A, thiamine, riboflavin, niacin, folate, and vitamin C and adequate intake was used for sodium and potassium. Logistic regression analysis was performed to estimate the odd ratios (ORs) and 95% confidential intervals (CIs) of the associations between micronutrient intakes and low handgrip strength. Multivariate analysis for covariates that may affect was performed after adjustment for age and sex as model 1. Model 2 added household income, current smoking status, monthly alcohol drinking, aerobic exercise, and muscular exercise to the model 1 covariates. Model 3 added comorbidities and energy intakes including total calorie, total protein, total fat and total carbohydrate to the model 2 covariates. Bold denotes *p* values < 0.05. *p* values < 0.05 were considered statistically significant. complex and vitamin C were significantly lower in older men with low handgrip strength, compared with the intake levels in the normal group. In women, there was a significant difference in the calcium, phosphorus, iron, potassium, riboflavin, niacin, folate, and vitamin C intake levels between groups. These findings are, in part, similar to those in the cross-sectional study conducted in 2019 by Beaudart et al., wherein a significantly increased prevalence of insufficiency was found for subjects with sarcopenia compared to those without sarcopenia in terms of potassium, magnesium, iron, calcium, vitamins E, and vitamins C intakes in a population with a mean age of 74.8 ± 5.9 years and consisting of 41.1% men and 58.9% women [40].

In logistic regression analysis after adjusting for all confounding factors, insufficient intake of potassium among the investigated micronutrients showed higher ORs for low handgrip strength in both men and women. A review study by Dronkelaar et al., which investigated the relationship between minerals and sarcopenia, demonstrated that the intakes of magnesium, selenium, calcium, and phosphorus were associated with the prevalence of sarcopenia in older adults [41]. A variety of studies have reported correlations between sarcopenia and vitamin intakes, including vitamin A [23], vitamin C [23,40], and folate [42]. Contradictory study has been reported no association between sarcopenia and vitamins such as vitamin B6, vitamin C, or folate [43]. This is presumably due to the difference between micronutrient intakes and diverse genetic or environmental variables such as race, body shape, consumed food patterns, measurement tools, and diagnosis criteria of muscle strength weakness related diseases such as dynapenia or sarcopenia among different regions and countries.

The relationship between sarcopenia and various nutrient factors, including selenium [43], zinc [44], vitamin D [45], long-chain polyunsaturated fatty acids, omega-3 [46,47], leucine [48], and β-hydroxy-β-methylbutyric acid [49,50], has been elucidated through numerous studies. Various mechanisms act in complicated ways during aging, and the relationship between nutritional intakes and age-related diseases or symptoms can be an important issue in the aging process, as many physical and functional parts are weakened. Through further comprehensive and elaborate cohort studies, it is needed to elucidate on intake amounts and the role of potassium in muscle strength weakness related diseases as potential interventional therapies for the prevention of dynapenia or sarcopenia.

The strength of this study is that it used a generalized dataset with systemically well-established national representative data obtained from the KNHANES, which involved a healthy community-dwelling study population in Korea. Since all examinations were conducted by professional staff in each field of investigation, the reliability of the original data and their interpretation is quite high. By using recently collected data, to our best knowledge, the current statuses of the representative Koreans reflect the associations between intake of minerals and vitamins and handgrip strength in older adults, following gender. 

Despite these strengths, this study had some limitations. Since handgrip strength is one of the methods for measuring muscle strength, it cannot fully represent diseases such as dynapenia or sarcopenia. Moreover, it is necessary to distinguish between sarcopenia and dynapenia and to analyze them using more validated and sophisticated tools that reflect muscle mass, strength, and physical performance. Furthermore, we analyzed data obtained using the widely used 24-h recall method for nutrient evaluation in the current study, which did not fully reflect the nutritional status of the participants. In the logistic regression analysis used to elucidate the association between micronutrient intake and low handgrip strength, the Korean dietary recommended nutrient intake or adequate intake values were compared as a reference; thus, this study should be interpreted as a reference that is limited to Koreans in terms of race. Since the KNHANES is a cross-sectional study, it is difficult to explain causal relationships.

## 5. Conclusions

In conclusion, potassium intake among micronutrients in Korean older populations with low handgrip strength might need continuous monitoring for the intervention or prevention of dynapenia or sarcopenia in clinical practice. The results of the present study may provide basic evidence to address the relationships between micronutrients such as potassium and muscle strength weakness, and aid in prevention of geriatric muscle decline. Further large-scale randomized controlled trials are essential to identify the association between micronutrients and muscle strength weakness related diseases such as dynapenia or sarcopenia.

## Figures and Tables

**Table 1:** Baseline characteristics of the participants with low handgrip strength or normal handgrip strength in men and women.

Variables	Men (*n* = 546)			Women (*n* = 708)		
	LHS (*n* = 103, 18.9%)	Normal (*n* = 443, 81.1%)	*p* Value	LHS (*n* = 168, 23.7%)	Normal (*n* = 540, 76.3%)	*p* Value
Age (years)	75.92 ± 0.46	72.07 ± 0.27	**<0.001**	75.27 ± 0.44	71.46 ± 0.24	**<0.001**
Household income, *n* (%)			**<0.001**			**0.001**
Low	60 (56.6)	163 (33.3)		113 (64.8)	253 (45.8)	
Middle-low	28 (30.8)	156 (35.8)		25 (14.4)	159 (27.8)	
Middle-high	12 (9.0)	76 (18.6)		20 (14.8)	89 (18.5)	
High	3 (3.6)	48 (12.3)		10 (6.0)	39 (7.9)	
Current smoking status, *n* (%)			0.213			0.631
No	80 (75.4)	366 (82.4)		164 (97.6)	526 (96.8)	
Yes	23 (24.6)	77 (17.6)		4 (2.4)	14 (3.2)	
Monthly alcohol consumption, *n* (%)			**<0.001**			0.080
No	61 (58.1)	151 (32.9)		146 (85.2)	426 (78.4)	
Yes	42 (41.9)	292 (67.1)		22 (14.8)	114 (21.6)	
Aerobic exercise, *n* (%)			**<0.001**			0.128
No	84 (83.2)	266 (59.8)		129 (75.7)	367 (67.6)	
Yes	19 (16.8)	177 (40.2)		39 (24.3)	173 (32.4)	
Muscular exercise (Strengthening), *n* (%)			**<0.001**			**0.034**
No	95 (90.7)	282 (64.5)		158 (94.0)	475 (87.4)	
Yes	8 (9.3)	161 (35.5)		10 (6.0)	65 (12.6)	
Handgrip strength (kg)	23.69 ± 0.38	36.10 ± 0.26	**<0.001**	15.03 ± 0.20	22.84 ± 0.18	**<0.001**
Height (cm)	162.79 ± 0.58	166.85 ± 0.30	**<0.001**	150.29 ± 0.45	153.57 ± 0.27	**<0.001**
Body weight (kg)	60.67 ± 1.02	67.26 ± 0.51	**<0.001**	53.23 ± 0.60	57.52 ± 0.42	**<0.001**
Body mass index (kg/m^2^)	22.88 ± 0.37	24.14 ± 0.16	**0.002**	23.52 ± 0.24	24.37 ± 0.17	**0.004**
Comorbidities, *n* (%)						
Hypertension			0.530			0.181
No	18 (19.7)	70 (14.6)		20 (10.4)	65 (13.5)	
Pre-stage	21 (19.3)	94 (22.6)		26 (17.5)	119 (22.8)	
Yes	64 (60.9)	279 (62.8)		122 (72.2)	356 (63.7)	
Obesity			0.053			**0.043**
Low-weight	8 (8.1)	12 (2.5)		8 (4.3)	10 (1.8)	
Normal	38 (38.0)	152 (33.3)		60 (37.0)	177 (33.8)	
Pre-stage	29 (29.2)	128 (29.7)		51 (31.9)	142 (26.8)	
Yes	28 (24.6)	151 (34.5)		49 (26.8)	211 (37.6)	
Diabetes			0.759			0.142
No	21 (18.4)	82 (18.2)		35 (21.0)	116 (22.3)	
Pre-stage	52 (54.4)	217 (50.8)		80 (45.1)	284 (52.3)	
Yes	30 (27.2)	144 (31.0)		53 (33.9)	140 (25.3)	
Hypercholesterolemia			0.775			0.337
No	75 (71.6)	310 (70.1)		94 (54.6)	280 (49.4)	
Yes	28 (28.4)	133 (29.9)		74 (45.4)	260 (50.6)	

Data are presented as mean ± standard error or percentage (*n* (%)). Bold denotes *p* values < 0.05. *p* values < 0.05 were considered statistically significant. LHS; low handgrip strength.

**Table 2:** Comparison of nutritional intake status of the participants in men and women.

Variables	Men (*n* = 546)			Women (*n* = 708)		
	LHS (*n* = 103, 18.9%)	Normal (*n* = 443, 81.1%)	*p* Value	LHS (*n* = 168, 23.7%)	Normal (*n* = 540, 76.3%)	*p* Value
Total calorie (kcal)	1552.26 ± 55.78	1884.10 ± 37.20	**<0.001**	1309.35 ± 41.76	1369.13 ± 22.56	0.153
Total protein (g)	54.47 ± 3.11	66.17 ± 1.80	**0.001**	43.36 ± 1.60	47.14 ± 1.04	**0.035**
(g/kg BM/day)	0.90 ± 0.04	1.00 ± 0.03	0.530	0.83 ± 0.03	0.83 ± 0.02	0.965
Total fat (g)	24.07 ± 1.77	35.38 ± 1.39	**<0.001**	23.84 ± 1.84	24.56 ± 0.82	0.703
(g/kg BM/day)	0.40 ± 0.03	0.53 ± 0.02	**<0.001**	0.46 ± 0.04	0.43 ± 0.01	0.516
Total carbohydrate (g)	256.40 ± 9.19	297.67 ± 4.86	**<0.001**	227.14 ± 7.67	237.72 ± 3.77	0.171
(g/kg BM/day)	4.29 ± 0.16	4.50 ± 0.09	0.227	4.38 ± 0.17	4.19 ± 0.07	0.276
Calcium (mg)	428.31 ± 31.58	558.28 ± 21.28	**<0.001**	350 ± 19.20	436.42 ± 14.45	**<0.001**
Phosphorus (mg)	856.38 ± 43.28	1058.29 ± 24.84	**<0.001**	693.32 ± 24.45	793.61 ± 17.26	**<0.001**
Iron (mg)	10.19 ± 0.60	13.19 ± 0.45	**<0.001**	8.12 ± 0.31	9.35 ± 0.21	**0.001**
Sodium (mg)	2889.15 ± 172.37	3445.53 ± 89.64	**0.003**	2234.78 ± 114.85	2350.50 ± 73.88	0.363
Potassium (mg)	2343.88 ± 147.37	2918.60 ± 77.12	**<0.001**	1904.16 ± 64.23	2410.76 ± 66.66	**<0.001**
Vitamin A (µgRAE)	266.69 ± 38.58	401.98 ± 40.28	**0.012**	277.48 ± 23.28	308.08 ± 17.93	0.237
β- carotenoids (µg)	2250.76 ± 366.10	3015.57 ± 178.38	**0.038**	2298.45 ± 189.45	2720.39 ± 189.47	0.094
Retinol (µg)	79.12 ± 13.22	150.68 ± 37.95	0.076	85.95 ± 15.76	81.23 ± 5.66	0.774
Thiamine (mg)	1.14 ± 0.06	1.34 ± 0.04	**0.002**	0.93 ± 0.03	0.99 ± 0.02	0.113
Riboflavin (mg)	1.09 ± 0.08	1.47 ± 0.05	**0.002**	0.92 ± 0.05	1.09 ± 0.04	**0.002**
Niacin (mg)	9.99 ± 0.64	12.12 ± 0.33	**0.003**	7.95 ± 0.30	8.87 ± 0.23	**0.011**
Folate (µgDFE)	302.75 ± 20.41	365.33 ± 10.33	**0.003**	248.43 ± 10.77	292.70 ± 8.02	**<0.001**
Vitamin C (mg)	46.69 ± 5.68	63.98 ± 3.61	**0.009**	50.84 ± 4.46	66.33 ± 4.73	**0.008**

Data are presented as mean ± standard error or percentage (*n* (%)). Bold denotes *p* values < 0.05. *p* values < 0.05 were considered statistically significant. LHS; low handgrip strength

**Table 3:** Multiple logistic regression for low handgrip strength in men aged ≥ 65 years.

Regression Models	Reference	Unadjusted			Model 1			Model 2			Model 3		
		OR	95% CI	*p* Value	OR	95% CI	*p* Value	OR	95% CI	*p* Value	OR	95% CI	*p* Value
Calcium intake (<700 mg)	≥700 mg	2.662	1.272–5.568	**0.010**	2.438	1.121–5.299	**0.025**	2.104	0.975–4.538	0.058	1.824	0.648–5.131	0.253
Phosphorus intake (<700 mg)	≥700 mg	2.752	1.676–4.519	**<0.001**	2.181	1.322–3.597	**0.003**	1.880	1.156–3.056	**0.011**	1.665	0.802–3.457	0.170
Iron intake (<9 mg)	≥9 mg	2.235	1.359–3.676	**0.002**	1.723	1.015–2.923	**0.044**	1.461	0.861–2.478	0.158	1.028	0.500–2.114	0.940
Sodium intake (≥1300 mg)	<1300 mg	1.439	0.712–2.907	0.308	1.304	0.663–2.564	0.440	0.945	0.481–1.855	0.868	0.732	0.318–1.688	0.462
Potassium intake (<3500 mg)	≥3500 mg	3.558	1.758–7.201	**0.001**	3.133	1.488–6.598	**0.003**	3.060	1.413–6.627	**0.005**	3.159	1.164–8.578	**0.024**
Vitamin A intake (<700 µg RAE)	≥700 µg RAE	2.848	1.029–7.882	**0.044**	2.739	1.034–7.255	**0.043**	2.905	1.121–7.524	**0.028**	2.564	0.834–7.877	0.100
Thiamine intake (<1.1 mg)	≥1.1 mg	2.882	1.680–4.944	**<0.001**	2.519	1.462–4.342	**0.001**	2.282	1.327–3.924	**0.003**	1.902	0.919–3.934	0.083
Riboflavin intake (<1.4 mg)	≥1.4 mg	2.555	1.466–4.454	**0.001**	2.096	1.161–3.784	**0.014**	1.702	0.936–3.096	0.081	1.213	0.456–3.225	0.697
Niacin intake (<14 mg)	≥14 mg	1.751	0.995–3.082	0.052	1.579	0.864–2.884	0.136	1.370	0.724–2.594	0.330	0.787	0.341–1.818	0.573
Folate intake (<400 µg DFE)	≥400 µg DFE	2.115	1.196–3.739	**0.010**	1.968	1.064–3.639	**0.031**	1.685	0.886–3.204	0.111	1.309	0.628–2.731	0.470
Vitamin C intake (<100 mg)	≥100 mg	2.185	0.920–5.191	0.076	2.096	0.919–4.782	0.078	1.690	0.738–3.870	0.213	1.212	0.494–2.971	0.673

Dietary intake was compared with the Korea Dietary Recommendation Intake criteria. Recommended intake was used for calcium, phosphorus, iron, potassium, vitamin A, thiamine, riboflavin, niacin, folate, and vitamin C and adequate intake was used for sodium and potassium. Logistic regression analysis was performed to estimate the odd ratios (ORs) and 95% confidential intervals (CIs) of the associations between micronutrient intakes and low handgrip strength. Multivariate analysis for covariates that may affect was performed after adjustment for age and sex as model 1. Model 2 added household income, current smoking status, monthly alcohol drinking, aerobic exercise, and muscular exercise to the model 1 covariates. Model 3 added comorbidities and energy intakes including total calorie, total protein, total fat and total carbohydrate to the model 2 covariates. Bold denotes *p* values < 0.05. *p* values < 0.05 were considered statistically significant.

## Data Availability

Publicly available datasets were analyzed in the current study and can be downloaded at https://knhanes.kdca.go.kr/knhanes (accessed on 1 January 2022).
